# Multiple Sclerosis With Migraine and Pyoderma Gangrenosum Treated With Ofatumumab and Erenumab

**DOI:** 10.7759/cureus.101886

**Published:** 2026-01-20

**Authors:** Haruhiko Motegi, Teppei Komatsu, Asako Onda, Kenichiro Sakai, Yasuyuki Iguchi

**Affiliations:** 1 Department of Neurology, The Jikei University School of Medicine, Tokyo, JPN

**Keywords:** erenumab, migraine, multiple sclerosis, ofatumumab, pyoderma gangrenosum

## Abstract

Multiple sclerosis (MS) is a chronic inflammatory demyelinating disease of the central nervous system. Migraine is a common comorbidity in MS, with primary headache syndromes frequently observed. Ofatumumab, a high-efficacy anti-cluster of differentiation 20 (CD20) monoclonal antibody, and erenumab, a calcitonin gene-related peptide (CGRP) receptor antagonist, are available as subcutaneously administered monoclonal antibody agents. Despite their independent efficacy, there is limited evidence regarding the concurrent use of these two agents in patients with MS and migraine, especially in those with dermatological conditions such as pyoderma gangrenosum. We treated a 43-year-old Japanese woman with relapsing-remitting MS (RRMS), migraine, and pyoderma gangrenosum. She experienced persistent migraines despite valproic acid and sumatriptan use. After the administration of intravenous methylprednisolone (IVMP), ofatumumab treatment was introduced; erenumab was later initiated to manage migraines. The addition of erenumab reduced the patient's monthly migraine days (MMDs) and monthly headache days (MHDs). No progression of MS or worsening of pyoderma gangrenosum was observed. The Expanded Disability Status Scale (EDSS) score remained stable, and cognitive function was preserved. The combination therapy demonstrated effectiveness without exacerbating the patient's underlying dermatological condition. This case suggests that combining ofatumumab and erenumab is a viable therapeutic option for patients with MS and comorbid migraine and pyoderma gangrenosum, offering effective disease control with an acceptable safety profile.

## Introduction

Multiple sclerosis (MS) is an inflammatory demyelinating disease of the central nervous system with an unknown cause. It most commonly presents as relapsing-remitting MS (RRMS). Headache is a common health concern in general medical practice, and primary headache syndromes are frequently observed in individuals with MS, with a prevalence estimated at 57%, predominantly comprising migraine without aura [1].

In Japan, eight disease-modifying therapies (DMTs) are approved for the prevention of relapses in MS. Ofatumumab, approved in the United States in 2020, is the only anti-cluster of differentiation 20 (CD20) monoclonal antibody available in Japan and is classified as a high-efficacy DMT [2]. Significant advances in the use of biologics for the treatment of migraine have been achieved, with agents targeting calcitonin gene-related peptide (CGRP) and its receptor (CGRP-R) [3-5]. Three such agents, erenumab, fremanezumab, and galcanezumab, have been approved in Japan [6].

While the combination of monoclonal antibody therapies with distinct mechanisms of action has been explored in oncology, evidence for such approaches in neurology remains scarce [7-9]. Despite the common coexistence of migraine in MS, there is no established evidence regarding the safety and efficacy of using these antibody therapies simultaneously for MS and migraine. In addition, all approved formulations of ofatumumab for MS and biologics for migraine are administered subcutaneously. Their safety in patients with dermatological conditions, particularly those with pyoderma gangrenosum associated with pathergy, has not been established.

We report the case of a patient with MS complicated by migraine and pyoderma gangrenosum. The patient was successfully treated with a combination of ofatumumab and erenumab, achieving control of both her MS and migraine without exacerbating her pyoderma gangrenosum.

## Case presentation

A 43-year-old Japanese woman with a history of migraine had been taking valproic acid (400 mg daily) for prophylaxis and sumatriptan (50 mg) during migraine attacks; however, she had trouble controlling her headaches, with monthly migraine days (MMDs) totaling 16 and monthly headache days (MHDs) reaching 30. Three years prior to her presentation, she developed blisters on her lower extremities and was diagnosed with pyoderma gangrenosum. Around the same time, she tested positive for anti-Sjögren's syndrome-related antigen A (SSA) antibodies and was diagnosed with Sjögren's syndrome.

**Figure 1:**
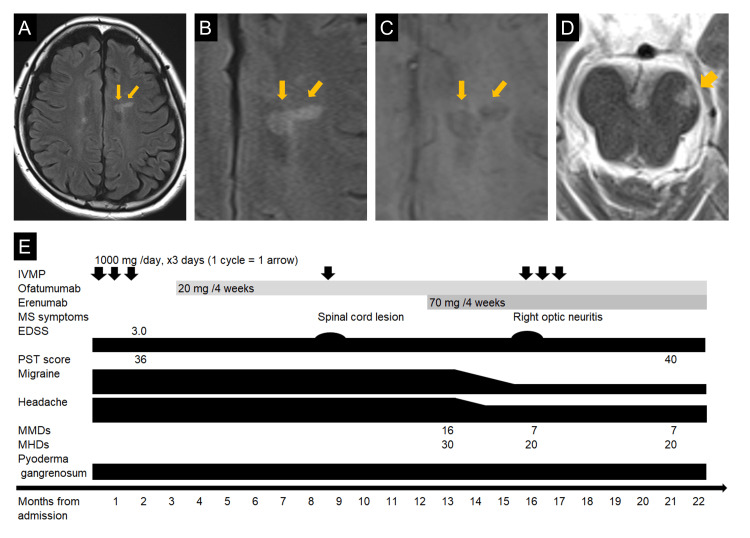
Brain MRI and Clinical Course An MRI examination of the patient, a 43-year-old woman, revealed a periventricular lesion and a paramagnetic rim lesion (indicated by arrows). The findings were observed on fluid-attenuated inversion recovery (A and B) and susceptibility-weighted imaging (C). A lesion involving the left cerebral peduncle was also identified (D). Throughout the clinical course, the patient's Expanded Disability Status Scale (EDSS) score and processing speed test (PST) score (E) did not deteriorate, and the number of her monthly migraine days (MMDs) and monthly headache days (MHDs) decreased IVMP, intravenous methylprednisolone; MS, multiple sclerosis

At the age of 43, she developed headaches, decreased vision in the left eye, numbness in the limbs, and urinary dysfunction, followed by the appearance of diplopia in winter. A brain MRI examination revealed left optic neuritis and multiple T2 hyperintense lesions around the periventricular area and brainstem, along with a paramagnetic rim lesion (PRL) (Figure 1A-1D), raising the suspicion of MS. A serum anti-aquaporin-4 (AQP4) antibody test using an enzyme-linked immunosorbent assay (ELISA) was negative. A cerebrospinal fluid analysis showed a cell count of six cells/µL (mononuclear cells: 83%) and an elevated protein level (101 mg/dL). The IgG index was 1.63, and 10 oligoclonal bands of type 2 were identified. Based on these findings, we diagnosed her with RRMS.

Ofatumumab (20 mg every four weeks) was introduced after three cycles of intravenous methylprednisolone (IVMP, 1000 mg/day for three consecutive days). Five months later, the patient reported numbness in her right foot, and a new lesion in the cervical spinal cord was detected on MRI. The numbness improved following one cycle of IVMP treatment. Four months later, erenumab (70 mg every four weeks) was initiated for the patient's migraines, reducing her MMDs from 16 to seven and her MHDs from 30 to 20. Three months after that, she reported abnormal color perception in her right eye, and an MRI evaluation suggested right optic neuritis. Her visual symptoms improved after three courses of IVMP.

Throughout the patient's clinical course (Figure 1E), her Expanded Disability Status Scale (EDSS) score remained 3.0; there was no evidence of relapse-associated worsening or progression, independent of relapse activity, and no decline in cognitive function as assessed by the processing speed test (PST) [10]. There was also no exacerbation of pyoderma gangrenosum.

The patient provided full, informed written consent for the publication of this report and images. The processing speed test (PST) is available for research use without special permission; thus, this case report did not utilize any assessment instruments subject to copyright or licensing constraints.

## Discussion

We diagnosed the patient with RRMS. Her case is representative of MS complicated by Sjögren's syndrome and pyoderma gangrenosum. Although the coexistence of autoimmune diseases in MS is relatively rare, it is not unprecedented in the Asian population [11]. In this patient's case, the central nervous system lesions met the criteria for dissemination in space and time, and the cerebrospinal fluid oligoclonal bands were positive, fulfilling the McDonald diagnostic criteria for MS. Some periventricular lesions were also identified as paramagnetic rim lesions (PRLs). PRLs reflect microglial activation specific to MS and support its diagnosis [12].

Combination therapy with two monoclonal antibody agents, each with distinct mechanisms of action, has been used in the field of oncology. For example, nivolumab is an antibody targeting programmed cell death protein 1, and ipilimumab is an antibody against cytotoxic T lymphocyte-associated antigen 4. The combination therapy of nivolumab and ipilimumab is used as a treatment regimen for advanced renal cell carcinoma and advanced non-small-cell lung cancer [7,8]. In another example, atezolizumab, an antibody targeting programmed cell death protein 1, and bevacizumab, an antibody against vascular endothelial growth factor, are applied as combination therapy for unresectable hepatocellular carcinoma [9].

There is limited evidence about the use of combination therapy with two monoclonal antibody agents in the field of neurology. Ofatumumab targets CD20, and erenumab is an antibody against CGRP-R. Our patient experienced mild relapses of MS after the initiation of ofatumumab and around the time that erenumab was introduced; however, her EDSS and PST scores remained stable without worsening overall. In addition, after the erenumab was introduced, the patient's MMD and MHD values both decreased. This outcome suggests that the combination of ofatumumab and erenumab did not diminish the therapeutic efficacy of either agent.

Data on the safety of monoclonal antibody agents in patients with dermatological conditions such as pyoderma gangrenosum exhibiting pathergy are limited. Fully human antibodies may play a key role in such cases. Ofatumumab and erenumab are fully human antibodies and are believed to induce less inflammation than humanized (ocrelizumab, fremanezumab, or galcanezumab) or chimeric (rituximab) antibodies [4,13]. Although direct comparative studies are lacking, it has been suggested that injection-site reactions with ofatumumab occur in approximately 12% of cases [13], which is lower compared to >30% with ocrelizumab [14] and >70% with rituximab [15]. Similarly, the rate of injection-site pain with erenumab is approximately 6% [4], showing a trend toward fewer reactions compared to fremanezumab or galcanezumab [3,5].

The single dose of ofatumumab used for MS is approximately 1/50th of the dose used for chronic lymphocytic leukemia (CLL). In the COMPLEMENT 1 study for CLL, the incidence of infusion-related reactions was 67%, which was higher than that observed in MS [16]. In our patient's case, combining ofatumumab and erenumab did not exacerbate her pyoderma gangrenosum. This result may be attributed to the fully human nature of these antibodies (which are less likely to trigger immune responses), along with the minimal doses used.

## Conclusions

We have described the case of a patient with MS complicated by migraine and pyoderma gangrenosum that was successfully managed with a combination of ofatumumab and erenumab, achieving control of both her MS and migraines without exacerbating her pyoderma gangrenosum. This case indicates that ofatumumab and erenumab may be safely used in patients with MS with comorbid migraines and pyoderma gangrenosum. Careful monitoring remains essential, and additional reports and prospective studies will help guide clinical practice.
